# Preoperative hemoglobin-platelet ratio can significantly predict progression and mortality outcomes in patients with T1G3 bladder cancer undergoing transurethral resection of bladder tumor

**DOI:** 10.18632/oncotarget.23896

**Published:** 2018-01-03

**Authors:** Gang Tang, Yunpeng Zhen, Wanqin Xie, Yinlei Wang, Feiran Chen, Chuan Qin, Han Yang, Zhiyong Du, Zhonghua Shen, Bo Zhang, Zhouliang Wu, Dawei Tian, Hailong Hu

**Affiliations:** ^1^ Department of Urology, The Second Hospital of Tianjin Medical University, Hexi District, Tianjin 300211, China; ^2^ Tianjin Key Laboratory of Urology, Tianjin Institute of Urology, The Second Hospital of Tianjin Medical University, Tianjin 300211, China; ^3^ Key Laboratory of Genetics and Birth Health of Hunan Province, The Family Planning Research Institute of Hunan Province, Changsha, Hunan 410126, China

**Keywords:** HPR, T1G3 bladder cancer, PFS, OS, CSS

## Abstract

**Objective:**

To investigate the prognostic role of hematological biomarkers, especially hemoglobin-platelet ratio (HPR) in the oncological outcomes in stage 1 and grade 3 (T1G3) bladder cancer.

**Materials and Methods:**

We identified 457 T1G3 bladder cancer patients who underwent transurethral resection of the bladder (TURB) between 2009 and 2014. Based on hematological parameters (hemoglobin-platelet ratio (HPR), hemoglobin, and platelet counts), recurrence-free survival (RFS), progression-free survival (PFS), and overall survival (OS) and cancer-specific survival (CSS) were analyzed by using Kaplan-Meier analysis. Multivariate Cox regression model was adopted to identify the predictors of oncological outcomes.

**Results:**

Kaplan-Meier survival analysis showed that low HPR (< 0.615), low hemoglobin (< 125g/l) and elevated platelet counts (> 240 × 10^3^/μl) were correlated with poor OS. Low HPR, but not low hemoglobin and high platelet counts, is associated with worse PFS. Low HPR and low hemoglobin, but not elevated platelet counts, are associated with worse CSS. However, no significant difference was observed in RFS according to any of these hematological markers. On multivariate analysis, low HPR (HR = 1.27, 95% CI = 0.81–1.75, *P* = 0.030), low hemoglobin (HR = 1.20, 95% CI = 0.79–1.84, *P* = 0.028) and elevated platelet counts (HR = 1.07, 95% CI = 0.72–1.32, *P* = 0.038) were significantly associated with OS. Low hemoglobin (HR = 1.08, 95% CI = 0.68–1.82, *P* = 0.041) was significantly linked with CSS. Particularly, low HPR was identified as an independent predictor of PFS (HR = 1.16, 95% CI = 0.97–1.49, *P* = 0.033) and CSS (HR = 1.14, 95% CI = 0.87–1.78, *P* = 0.029).

**Conclusions:**

Preoperative HPR can be taken into account as a factor predictive of oncological outcomes for T1G3 bladder cancer, particularly disease progression and mortality outcomes.

## INTRODUCTION

Bladder cancer (BC) ranks as the ninth most frequently-diagnosed cancer worldwide, with an estimation of 430, 000 new cases diagnosed in 2012 [1]. Stage 1 and grade 3 (T1G3) bladder cancer is pathologically classified into non-muscle invasive bladder cancer (NMIBC), which is characterized by a high risk of recurrence and progression after the treatment of transurethral resection of bladder tumor (TURB) alone, with a recurrence rate of 50% to 70% and a tumor progression rate of 25% to 50% [2]. Due to its high propensity to recur and progress to muscle invasive disease, T1G3 BC is considered as the most challenging form of NMIBC, and reportedly, its long-term death rate can be as high as 34% [3]. Therefore, there is a clear need for predictive markers of recurrence, progression and survival in patients with T1G3 BC. In the past two decades, many biomarkers, for example, the gene expression signatures of patients, have been proposed for oncologic outcomes prediction in BC [4, 5]. However, the power of these predictive biomarkers is still insufficient to meet the clinic needs.

Recently, there has been increasing interest in the prognostic role of hematologic biomarkers in patients undergoing TURB. Neutrophil-lymphocyte ratio (NLR) has been repeatedly reported as an efficient biomarker to predict oncologic outcomes in patient undergoing TURB for NMIBC [6, 7]. Other hematologic biomarkers, including lymphocyte-monocyte ratio (LMR), platelet-lymphocyte ratio (PLR), have also been suggested [7, 8]. While attention is mainly focused on biomarkers that reflect the interaction between systemic inflammatory response and tumor, to date, the prognostic role of hemoglobin-platelet ratio has not been studied in T1G3 BC. Its consistency and significance as prognosticator are still unclear in T1G3 BC.

In this study, we aim to determine whether HPR is an independent predictor of disease progression, OS and CSS in T1G3 BC patients who underwent TURB.

## RESULTS

### Clinicopathological characteristics of patients

**Figure 1:**
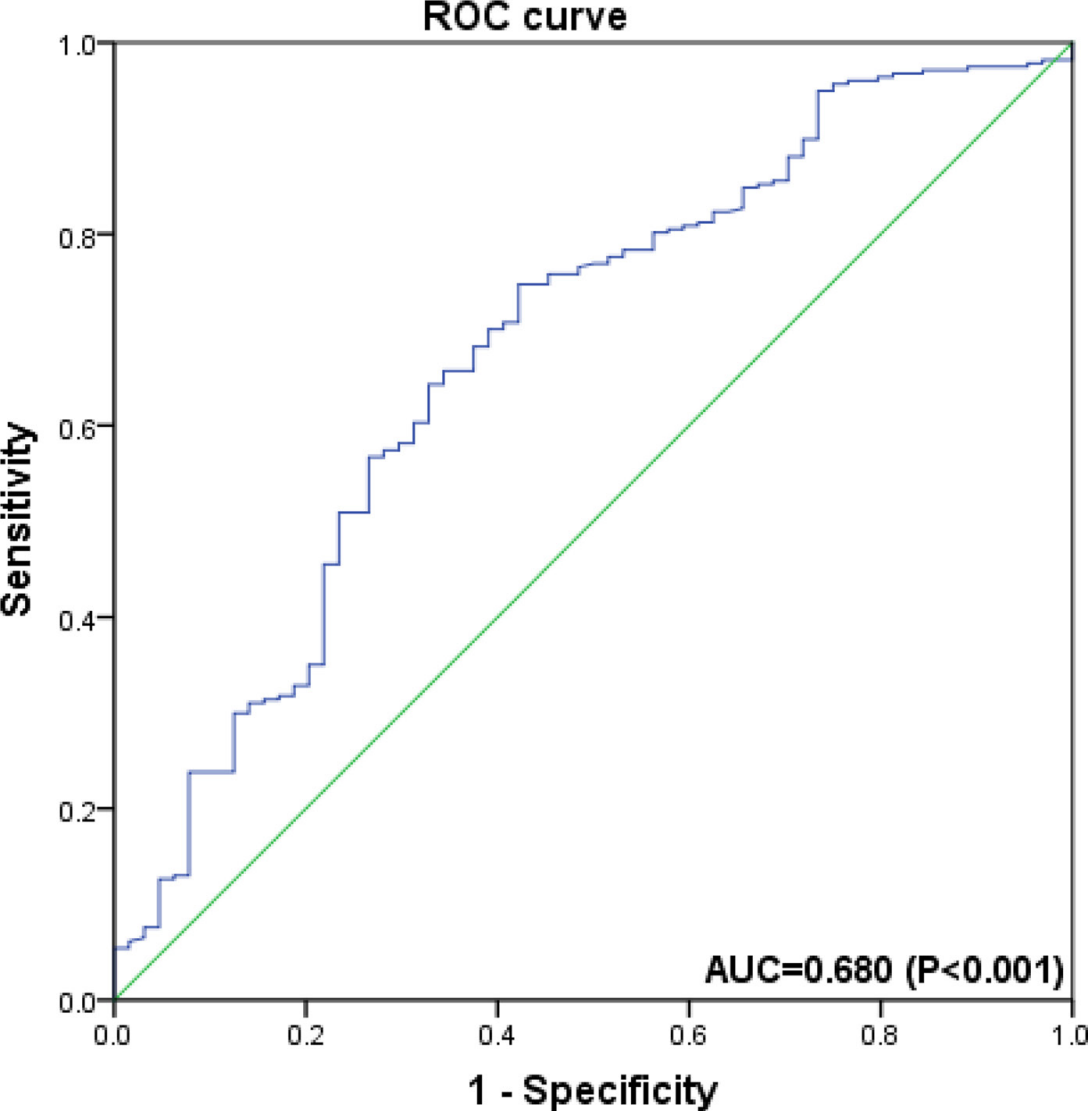
Receiver operating characteristic curve analysis of the ability of hemoglobin-platelet ratio (HPR) to discriminate for overall survival in overall population of T1G3 bladder cancer patients It suggests that HPR predicts OS with a sensitivity of 74.7% and a specificity of 57.8% (AUC = 0.680; 95% CI = 0.605–0.755; *P* < 0.001).

Data of the clinicopathological variables of the patients are summarized in Table 1. The mean follow-up for patients in this retrospective study was 39 months (IQR: 16-49). Of the 457 patients, 27.4% (*n* = 125) patients had cancer recurrence, and 9.6% (*n* = 44) progressed after initial TURB. The number of all-cause and cancer-specific death were 86 (18.8%) and 51 (11.2%), respectively. The median values of hematological markers were 131 for hemoglobin (IQR: 117-148), 232 for platelet counts (IQR: 188-316), and 0.625 for HPR (IQR: 0.283-0-802), respectively. The optimal cut-off value of HPR, which was 0.615 (Figure 1), was determined by receiver operating characteristic (ROC) curve. Similarly, the cut-off values of hemoglobin, platelet counts, NLR, PLR and LMR were 125, 240, 2.3, 128 and 3.2, respectively. According to the preoperative HPR status (< 0.615 versus ≥ 0.615), patients in the cohort were dichotomized. As shown in Table 2, no significant differences in the demographic variables including sex, smoking history, BMI, DM and hypertension were observed between the two groups (All *P* > 0.05). Furthermore, there were no primary differences in number of tumor, tumor size, NLR, PLR, LMR between the two groups (All *P* > 0.05).

**Table 1:** Clinicopathological variables of the 457 patients with T1G3 bladder cancer in the study

Variables	
Age, years	66 (54–74)
Gender, *n* (%)	
Male	355 (77.7)
Female	102 (22.3)
Smoking history, *n* (%)	171 (37.4)
BMI, kg/cm^2^	25.3 (21.8–27.5)
DM, *n* (%)	88 (19.3)
Hypertension, *n* (%)	220 (48.1)
Multifocal, *n* (%)	
Yes	185 (40.5)
No	272 (59.5)
Tumor size (cm), *n* (%)	
< 3	322 (70.5)
≥ 3	135 (29.5)
Oncological outcomes, *n* (%)	
Recurrence	125 (27.4)
Progression	44 (9.6)
All-cause mortality	86 (18.8)
Cancer-specific mortality	51 (11.2)
Hematological markers, *n* (%)	
Hemoglobin, g/l	131 (117–148)
Platelet counts, ×10^3^/μl	232 (188–316)
HPR	0.625 (0.283–0.802)
NLR	1.96 (1.45–2.68)
PLR	121.6 (89.7–192.4)
LMR	2.98 (2.33–3.87)
Follow-up duration (mon)	39 (16–49)

Abbreviations: T1G3, stage 1 and grade 3; BMI, body mass index; DM, diabetes mellitus; HPR, hemoglobin-to-platelet ratio; NLR, neutrophil-to-lymphocyte ratio; PLR, platelet-to-lymphocyte ratio; LMR, lymphocyte-to-monocyte ratio.

**Table 2:** Comparing the clinicopathological variables according to the preoperative HPR status in the 457 patients with T1G3 bladder cancer

Variables	HPR ≥ 0.615 (n = 277)	HPR < 0.615 (n = 180)	*P* value
Age, years	59 (52–65)	68 (59–73)	< 0.001
Gender, n (%)			
Male	217 (78.3)	138 (76.7)	0.675
Female	60 (21.7)	42 (23.3)	
Smoking history, n (%)			
Yes	94 (33.9)	77 (42.8)	0.056
No	183 (66.1)	103 (57.2)	
BMI, kg/cm^2^	24.3 (22.5–26.7)	25.8 (22.9–26.6)	0.069
DM, n (%)			
Yes	46 (16.6)	42 (23.3)	0.075
No	231 (83.4)	138 (76.7)	
Hypertension, n (%)			
Yes	126 (45.5)	94 (52.2)	0.159
No	151 (54.5)	86 (47.8)	
Multifocal, n (%)			
Yes	101 (36.5)	74 (41.1)	0.318
No	176 (63.5)	106 (58.9)	
Tumor size (cm), n (%)			
< 3	202 (72.9)	120 (66.7)	0.152
≥ 3	75 (27.1)	60 (33.3)	
Oncological outcomes, n (%)			
Recurrence	67 (24.2)	58 (32.2)	0.060
Progression	19 (6.9)	25 (13.9)	0.013
All-cause mortality	38 (12.3)	48 (29.9)	< 0.001
Cancer-specific mortality	22 (7.9)	29 (16.1)	0.008
Hematological marker, n (%)			
Hemoglobin (g/l)	138 (121–157)	121 (103–136)	0.016
Platelet counts (×10^3^/μl)	221 (183–274)	237 (190–306)	0.058
HPR	0.657 (0.304 – 0.864)	0.562 (0.237–0.715)	< 0.001
NLR	1.92 (1.37–2.47)	2.06 (1.58–2.76)	0.189
PLR	116 (81.3–179.6)	127 (94.3–202.5)	0.273
LMR	3.11 (2.52–3.96)	2.82 (2.21–3.74)	0.481
Follow-up duration (mon)	37 (14 – 43)	40 (17–45)	0.146

Abbreviations: T1G3, stage 1 and grade 3; BMI, body mass index; DM, diabetes mellitus; HPR, hemoglobin-to-platelet ratio; NLR, neutrophil-to-lymphocyte ratio; PLR, platelet-to-lymphocyte ratio; LMR, lymphocyte-to-monocyte ratio.

### Correlation between hematological markers and prognosis of the patients with T1G3 bladder cancer

**Figure 2:**
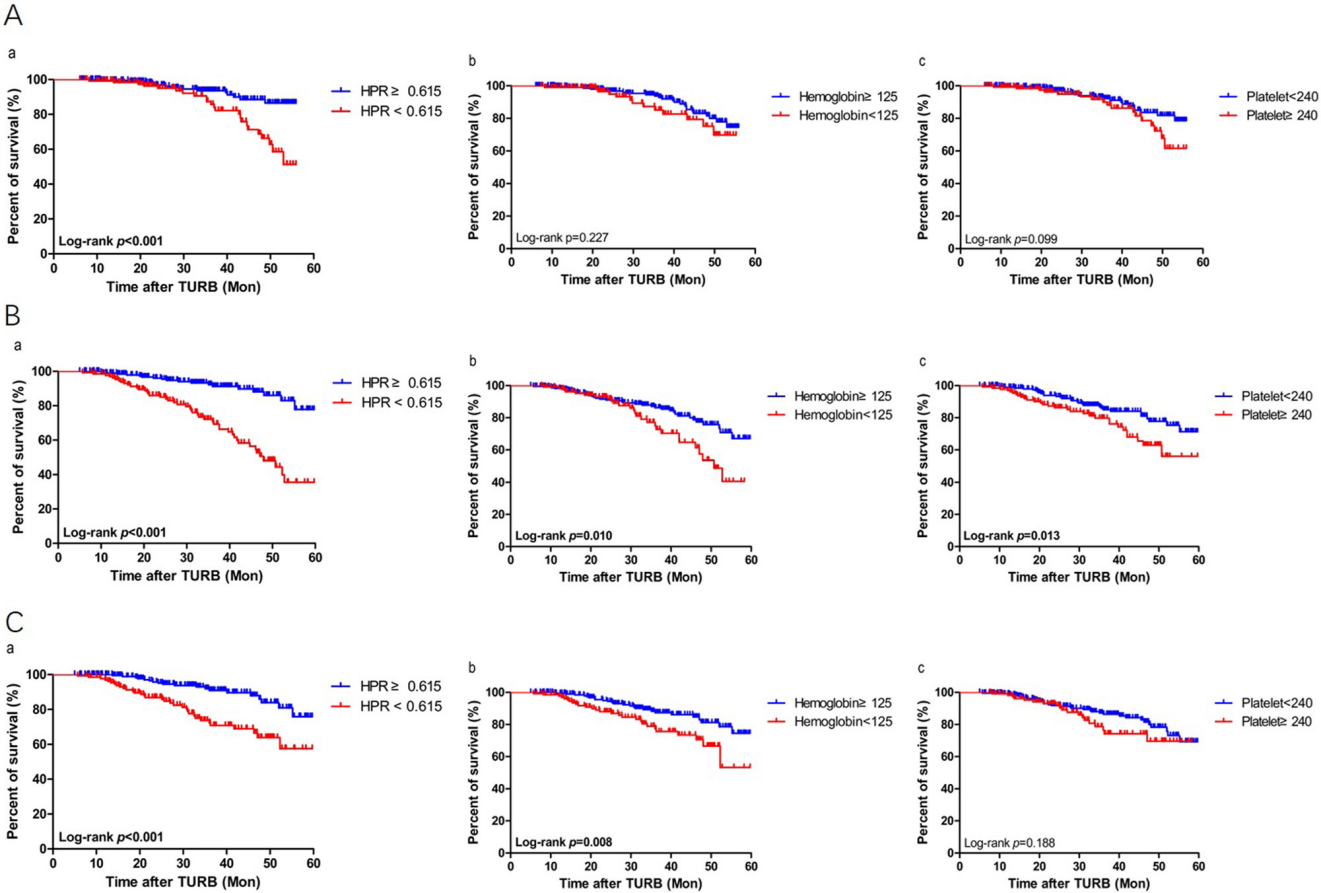
Kaplan-Meier survival estimates for comparing (**A**) progression-free, (**B**) overall and (**C**) cancer specific survivals according to the preoperative status of (a) hemoglobin-platelet ratio (HPR), (b) hemoglobin, and (c) platelet counts, respectively in the stage 1 and grade 3 (T1G3) bladder cancer patients who underwent transurethral resection of the bladder (TURB). Statistical differences between the two groups were compared by using the log-rank test.

We used Kaplan-Meier survival analysis to identify the associations of hematological markers (hemoglobin, platelet counts and HPR) with oncological outcomes. Notably, low HPR < 0.615) is associated with worse PFS, but not low hemoglobin (< 125) and elevated platelet counts (> 240) (Figure 2A). Moreover, low HPR, low hemoglobin and elevated platelet counts were significantly associated with poor OS (Figure 2B). In addition, low HPR and low hemoglobin, but not elevated platelet counts, were independently associated with poor CSS estimates in the cohort (Figure 2C). Interestingly, no significant difference was found in RFS rates according to hemoglobin, platelet counts or HPR (Supplementary Figure 1), and either in RFS, PFS, OS or CSS rates in terms of the hematologic biomarker NLR, PLR or LMR (data not shown).

### The primary predictors of progression and survival outcomes

We furthermore performed multivariate Cox regression analysis to identify the predictors of oncological outcomes in patients with T1G3 bladder cancer who underwent TURB. In univariate analysis, NLR, PLR and LMR showed marginal associations with PFS, OS and CSS, however, not statistically significant. The multivariate analysis showed that low HPR was a predictor for OS (HR = 1.27, 95% CI 0.81–1.75), and so was the age (HR = 1.24, 95% CI 1.03–1.43), low hemoglobin (HR = 1.20, 95% CI 0.79–1.84), and elevated platelet counts (HR = 1.07, 95% CI 0.72–1.32). The predictive ability of HPR for OS was also supported by ROC curve (AUC = 0.680; 95% CI = 0.605–0.755; *P* < 0.001). The multivariate analysis also revealed that low hemoglobin was a significant predictor for CSS (HR = 1.08, 0.68–1.82). More importantly, low HPR was identified as an independent predictor for PFS (HR = 1.16, 0.97–1.49) and CSS (HR = 1.14, 0.87–1.78), indicating that decease in HPR would increase the risk of progression and cancer-specific mortality (Table 3).

**Table 3:** Univariate and multivariate analyses for PFS, OS and CSS according to clinicopathological variables in T1G3 bladder cancer patients underwent TURB

Variables	Univariate			Multivariate		
	HR	95% CI	P	HR	95% CI	P
**PFS**						
Hemoglobin (g/l)						
≥ 125	1			1		
< 125	1.08	0.70–1.87	0.037	1.03	0.57–2.14	0.290
Platelet counts (×10^3^/μl)						
< 240	1			1		
≥ 240	1.21	0.78–1.47	0.016	1.02	0.65–1.53	0.110
**HPR**						
≥ 0.615	1			1		
< 0.615	1.31	1.02–1.46	0.003	**1.16**	0.97–1.49	**0.033**
NLR	1.16	0.96–1.32	0.053	1.11	0.91–1.47	0.131
PLR	1.04	0.82–1.28	0.061	1.17	0.74–1.33	0.461
LMR	1.09	0.87–1.29	0.073	1.24	0.62–1.56	0.533
**OS**						
**Age (year)**						
< 65	1			1		
**≥ 65**	1.25	1.10–1.29	< 0.001	**1.24**	1.03–1.43	**0.026**
**Hemoglobin (g/l)**						
≥ 125	1			1		
**< 125**	1.28	0.89–1.69	0.007	**1.20**	0.79–1.84	**0.028**
**Platelet counts (×10^3^/μl)**						
< 240	1			1		
**≥ 240**	1.18	0.81–1.33	0.009	**1.07**	0.72–1.32	0.038
**HPR**						
≥ 0.615	1			1		
**< 0.615**	1.22	0.93–1.61	0.002	**1.27**	0.81–1.75	**0.030**
NLR	2.13	1.43–2.89	0.059	1.83	1.13–3.02	0.241
PLR	1.85	1.21–2.67	0.066	1.45	1.03–2.84	0.378
LMR	1.63	0.88–2.04	0.070	1.02	0.64–2.29	0.409
CSS						
Hemoglobin (g/l)						
≥ 125	1			1		
**< 125**	1.32	1.08–1.69	0.004	**1.08**	0.68–1.82	**0.041**
Platelet counts (×10^3^/μl)						
< 240	1			1		
≥ 240	1.14	0.68–1.51	0.031	1.06	0.31–2.13	0.083
**HPR**						
≥ 0.615	1			1		
< 0.615	1.23	1.04–1.47	< 0.001	**1.14**	0.87–1.78	**0.029**
NLR	2.37	1.87–2.89	0.056	1.99	1.53–2.97	0.190
PLR	2.02	1.72–2.59	0.063	1.89	1.44–2.76	0.177
LMR	1.88	1.42–2.47	0.067	1.55	1.31–2.55	0.216

Abbreviations: PFS, progression-free survival; OS, overall survival; CSS, cancer-special survival; TURB, transurethral resection of bladder tumor; HR, hazard ratio; CI, confidence interval; T1G3, stage 1 and grade 3; HPR, hemoglobin-to-platelet ratio; NLR, neutrophil-to-lymphocyte ratio; PLR, platelet-to-lymphocyte ratio; LMR, lymphocyte-to-monocyte ratio.

## DISCUSSION

Recently, there has been increasing interest in the prognostic role of hematologic biomarkers in patients with bladder cancer. NLR has been highlighted as an efficient biomarker to predict oncologic outcomes in patients with bladder cancer [9–11]. Other biomarkers, including LMR and PLR, have also been reported [12, 13]. As a matter of fact, most of the biomarkers take advantage of the leukocyte parameters, and the prognostic role of hemoglobin level and platelet count have not been clearly defined. However, the number of studies exploring the prognostic role of hemoglobin and platelet level has been increasing [12, 14–18]. Previously, Sejima *et al*. [19] reported that hemoglobin level was an independent predictor of CSS (HR = 0.81). Grimm *et al*. [8] also observed that hemoglobin (< 13.4g/dl) was an independent prognostic parameter regarding OS and CSS (HR = 0.60, HR = 0.60). In a study by Hara *et al*. which enrolled 254 patients with bladder cancer who underwent RC, hemoglobin concentration was shown to be significantly associated with OS (HR = 0.93, Age < 75; HR = 8.61, Age > 75). Most recently, a meta-analysis involved 6 independent studies with 4447 patients provided the data showing that a higher hemoglobin level was associated with decreased disease (DR), all-cause mortality (ACM) and cancer-specific mortality (CSM) (HR = 0.95, HR = 0.90, HR = 0.90) [20]. With regard to platelet count, Moschini *et al*. [21] revealed that platelet count was significantly related to OS (HR = 1.64).

Although much attention has been drawn to the prognostic role of hemoglobin and platelet level in bladder cancer, few studies evaluated the performance of these biomarkers in patients with NMIBC, particularly T1G3 bladder cancer who underwent TURB. Further, there had been no report focusing on the relationship between preoperative HPR and prognosis of T1G3 bladder cancer patients. Thus, in the present study, we examined the associations of a set of hematologic markers (hemoglobin, platelet count and HPR) with the survival outcomes in a cohort consisting of 457 patients with T1G3 bladder cancer who underwent TURB. We show that preoperative HPR and hemoglobin level are independently associated with OS and CSS. Moreover, HPR is significantly related to PFS, and preoperative HPR is an independent prognostic factor indicating progression and mortal outcome in T1G3 bladder cancer patients undergoing TURB. NLR, LMR and PLR are well-characterized prognostic biomarkers in patients with bladder cancer. However, in our cohort, NLR, LMR and PLR showed no significant associations with survival outcomes when patients were grouped based on HPR status. More independent and multicenter-based studies are anticipated to access the prognostic value of the lymphocyte-related parameters in T1G3 bladder cancer.

With the finding of concomitant anemia and thrombocytosis in cancer patients, the underlying molecular mechanisms are also being investigated. Gakis and his colleagues observed that the degree of hematological disorders was often associated with more aggressive diseases [22]. One possible mechanism for this observation is that growing tumors induce thrombocytosis by secretion of growth factors and cytokines. Interleukin-6 (IL-6), which can be produced by malignant tumors, is able to stimulate platelet production. IL-6 and tumor necrosis factor-α (TNF-α) are also important cytokines for the development of tumor-induced anemia. These two cytokines may play a role in shortening erythrocyte half-life and reducing iron utilization and Epo production [23]. GATA-2, which is a transcription factor in hematopoietic progenitor cells and usually acts via interaction with GATA-1, is overexpressed in early immature hematopoietic progenitors to ensure their maintenance and proliferation. Overexpression of GATA-2 also can lead to increased platelet production and inhibition of erythrocyte differentiation [24, 25]. In tumor microenvironment, the increased platelets can affect angiogenesis by releasing vascular endothelial growth factor (VEGF) [26], which plays an important role in the progression of bladder cancer [27]. Besides, the aggregation of tumor cells with platelets can protect them from the natural killer (NK) cells of the host immune system [28], and platelets promote the adhesion of tumor cells to endothelial cells, which is a necessary step in the process of extravasation [29]. In addition to the increased platelet production, cancer–associated anemia causes hypoxia and induces upregulation of hypoxia-inducible factor-1α. This leads to the upregulation of genes involved in angiogenesis which inhibit apoptosis [30] and increase cancer cell transmission [31]. Moreover, anemia stimulates the release of VEGF [32] which promotes angiogenesis and increases the biological invasiveness of the tumor. Taken together, multiple lines of evidence suggest a profound impact of cancer-associated anemia and thrombocytosis in tumor development, and also implicate a link between the hematologic biomarkers and cancer prognosis.

Certainly, we are aware of the potential limitations that are associated with our retrospective study at a single center, for example, a potential selection bias on patients. In this study, we mainly focused on the preoperative hematologic markers. Nonetheless, these markers may not fully represent the dynamic changes of HPR. Further, the cut-off values of preoperative hematologic markers were adopted based on ROC curve analysis, and we realize that our cut-off values are not consistent with those of other studies. We speculate that the different cut-off values may reflect the inherent differences among those independent study populations, such as distinct genetic and environment backgrounds.

In conclusion, we have identified that preoperative HPR is significantly associated with PFS, OS and CSS in T1G3 bladder cancer patients who underwent TURB. Our study indicates that preoperative HPR can be taken into account as a factor predictive of oncological outcomes for T1G3 bladder cancer, particularly disease progression and mortality outcomes.

## MATERIALS AND METHODS

We retrospectively reviewed 457 cases in which patients were diagnosed with T1G3 bladder cancer and underwent TURB from April 2009 to December 2014 at the Second Hospital of Tianjin Medical University. Pathological staging and grading of each tumor were determined according to the International Society of Urological Pathology 1998/World Health Organization 2004 classification. All patients were followed up retrospectively through hospital records and telephone interviews with either the patients or their close relatives. Histological examination was evaluated by two experienced genitourinary pathologists in our institute. All patients were initially treated with TURB. Tumor was pathologically diagnosed as T1G3 and with no carcinoma *in situ*. And all serum parameters were obtained from the routine preoperative test results of the patients. We excluded patients with a short-term follow up period (less than six months after TURB) and those with systemic inflammation.

This study was conducted in accordance to the Declaration of Helsinki and its amendments and approved by the local Ethics Committee of the Second Hospital of Tianjin Medical University.

We collected clinical and pathological data, which included age, gender, smoking history, body mass index (BMI), diabetes mellitus (DM), hypertension, hemoglobin (g/l), platelet counts (×10^3^/μl), preoperative HPR, the number and size of tumors, and the oncological outcomes including recurrence, progression, all-cause mortality and cancer-specific mortality. The HPR was calculated based on the hemoglobin and platelet counts (HPR = hemoglobin/platelet counts). The cut-off values of the HPR, hemoglobin and platelet counts were determined with receiver operating characteristic (ROC) curve with the best accuracy (the greatest sensitivity and specificity) [33]. Median and interquartile range (IQR) were calculated for continuous variables.

Urine cytology and cystoscopy were performed at 3-months interval for a period of the first 2 years after the initial TURB. Then, cystoscopy was conducted semiannually until the fifth year and annually thereafter. Bladder biopsy was conducted when necessary. Computed tomography scan was performed every year to assess the status of patients. During the follow-up, the end point of patients in the study was the time when bladder cancer recurrence and tumor progression were histologically confirmed, and the time of dead and the latest visit.

### Statistical analysis

The outcome measures were RFS, PFS, OS and CSS evaluated in months from the date of TURB. The Kaplan-Meier survival analysis was conducted to evaluate progression and survival outcomes, and differences of the curves were examined by log-rank test. Univariate and multivariate analyses were performed using the Cox proportional hazards model to determine the independent predictors of various oncological outcomes. All statistical analysis was performed using SPSS 22.0 version statistical software (IBM, Armonk, New York, USA) and GraphPad Prism version 5.0 (GraphPad Software Inc., San Diego, CA, USA). The two-sided *p*-value < 0.05 indicates a statistically significant difference.

## SUPPLEMENTARY MATERIALS FIGURES



## Abbreviations

HPR: Hemoglobin-platelet ratio
T1G3: Stage 1 and grade 3
BC: Bladder cancer
TURB: Transurethral resection of the bladder
NMIBC: Non-muscle invasive bladder cancer
NLR: Neutrophil-lymphocyte ratio
LMR: Lymphocyte-monocyte ratio
PLR: Platelet-lymphocyte ratio
BMI: Body mass index
DM: Diabetes mellitus
ROC: Receiver operating characteristic
AUC: Areas under curve
IQR: Interquartile range
RFS: Recurrence-free survival
PFS: Progression-free survival
OS: Overall survival
CSS: Cancer-special survival
HR: Hazard ratio
CI: Confidence interval
DR: Decreased disease
ACM: All-cause mortality
CSM: Cancer-specific mortality
IL-6: Interleukin-6
TNF-α: Tumor necrosis factor-α
Epo: Erythropoietin
GATA-2: GATA binding protein 2
VEGF: Vascular endothelial growth factor
NK: Natural killer.

## References

[1] Antoni S, Ferlay J, Soerjomataram I, Znaor A, Jemal A, Bray F (2017). Bladder Cancer Incidence and Mortality: A Global Overview and Recent Trends. Eur Urol.

[2] Pham HT, Soloway MS (1997). High-risk superficial bladder cancer: intravesical therapy for T1 G3 transitional cell carcinoma of the urinary bladder. Semin Urol Oncol.

[3] Cookson MS, Herr HW, Zhang ZF, Soloway S, Sogani PC, Fair WR (1997). The treated natural history of high risk superficial bladder cancer: 15-year outcome. J Urol.

[4] Dyrskjøt L, Zieger K, Real FX, Malats N, Carrato A, Hurst C, Kotwal S, Knowles M, Malmström PU, de la Torre M, Wester K, Allory Y, Vordos D (2007). Gene expression signatures predict outcome in non-muscle-invasive bladder carcinoma: a multicenter validation study. Clin Cancer Res.

[5] Mengual L, Burset M, Ars E, Lozano JJ, Villavicencio H, Ribal MJ, Alcaraz A (2009). DNA microarray expression profiling of bladder cancer allows identification of noninvasive diagnostic markers. J Urol.

[6] Mano R, Baniel J, Shoshany O, Margel D, Bar-On T, Nativ O, Rubinstein J, Halachmi S (2015). Neutrophil-to-lymphocyte ratio predicts progression and recurrence of non-muscle-invasive bladder cancer. Urol Oncol.

[7] Kang M, Jeong CW, Kwak C, Kim HH, Ku JH (2017). Preoperative neutrophil-lymphocyte ratio can significantly predict mortality outcomes in patients with non-muscle invasive bladder cancer undergoing transurethral resection of bladder tumor. Oncotarget.

[8] Lee SM, Russell A, Hellawell G (2015). Predictive value of pretreatment inflammation-based prognostic scores (neutrophil-to-lymphocyte ratio, platelet-to-lymphocyte ratio, and lymphocyte-to-monocyte ratio) for invasive bladder carcinoma. Korean J Urol.

[9] Celik O, Akand M, Keskin MZ, Yoldas M, Ilbey YO (2016). Preoperative neutrophil-to-lymphocyte ratio (NLR) may be predictive of pathologic stage in patients with bladder cancer larger than 3 cm. Eur Rev Med Pharmacol Sci.

[10] Kaynar M, Yıldırım ME, Badem H, Caviş M, Tekinarslan E, Istanbulluoğlu MO, Karataş OF, Çimentepe E (2014). Bladder cancer invasion predictability based on preoperative neutrophil-lymphocyte ratio. Tumour Biol.

[11] Morizawa Y, Miyake M, Shimada K, Hori S, Tatsumi Y, Nakai Y, Anai S, Tanaka N, Konishi N, Fujimoto K (2016). Neutrophil-to-lymphocyte ratio as a detection marker of tumor recurrence in patients with muscle-invasive bladder cancer after radical cystectomy. Urol Oncol.

[12] Can C, Baseskioglu B, Yılmaz M, Colak E, Ozen A, Yenilmez A (2012). Pretreatment parameters obtained from peripheral blood sample predicts invasiveness of bladder carcinoma. Urol Int.

[13] Zhang GM, Zhu Y, Luo L, Wan FN, Zhu YP, Sun LJ, Ye DW (2015). Preoperative lymphocyte-monocyte and platelet-lymphocyte ratios as predictors of overall survival in patients with bladder cancer undergoing radical cystectomy. Tumour Biol.

[14] Grimm T, Buchner A, Schneevoigt B, Kretschmer A, Apfelbeck M, Grabbert M, Jokisch JF, Stief CG, Karl A (2016). Impact of preoperative hemoglobin and CRP levels on cancer-specific survival in patients undergoing radical cystectomy for transitional cell carcinoma of the bladder: results of a single-center study. World J Urol.

[15] Hara T, Matsuyama H, Kamiryo Y, Hayashida S, Yamamoto N, Nasu T, Joko K, Baba Y, Suga A, Yamamoto M, Aoki A, Takai K, Yoshihiro S, Yamaguchi Uro-Onocology Group (2016). Use of preoperative performance status and hemoglobin concentration to predict overall survival for patients aged ≥ 75 years after radical cystectomy for treatment of bladder cancer. Int J Clin Oncol.

[16] Chalfin HJ, Liu JJ, Gandhi N, Feng Z, Johnson D, Netto GJ, Drake CG, Hahn NM, Schoenberg MP, Trock BJ, Scott AV, Frank SM, Bivalacqua TJ (2016). Blood Transfusion is Associated with Increased Perioperative Morbidity and Adverse Oncologic Outcomes in Bladder Cancer Patients Receiving Neoadjuvant Chemotherapy and Radical Cystectomy. Ann Surg Oncol.

[17] Gershman B, Moreira DM, Tollefson MK, Frank I, Cheville JC, Thapa P, Tarrell RF, Thompson RH, Boorjian SA (2016). The association of ABO blood type with disease recurrence and mortality among patients with urothelial carcinoma of the bladder undergoing radical cystectomy. Urol Oncol.

[18] Moschini M, Bianchi M, Rossi MS, Dell Oglio P, Gandaglia G, Fossati N, Mattei A, Damiano R, Shariat SF, Salonia A, Montorsi F, Briganti A, Colombo R, Gallina A (2016). Timing of blood transfusion and not ABO blood type is associated with survival in patients treated with radical cystectomy for nonmetastatic bladder cancer: results from a single high-volume institution. Urologic Oncology: Seminars and Original Investigations.

[19] Sejima T, Morizane S, Yao A, Isoyama T, Saito M, Amisaki T, Koumi T, Takenaka A (2014). Prognostic impact of preoperative hematological disorders and a risk stratification model in bladder cancer patients treated with radical cystectomy. Int J Urol.

[20] Xia L, Guzzo TJ (2017). Preoperative Anemia and Low Hemoglobin Level Are Associated With Worse Clinical Outcomes in Patients With Bladder Cancer Undergoing Radical Cystectomy: A Meta-Analysis. Clin Genitourin Cancer.

[21] Moschini M, Suardi N, Pellucchi F, Rocchini L, La Croce G, Capitanio U, Briganti A, Damiano R, Montorsi F, Colombo R (2014). Impact of preoperative thrombocytosis on pathological outcomes and survival in patients treated with radical cystectomy for bladder carcinoma. Anticancer Res.

[22] Gakis G, Todenhöfer T, Stenzl A (2011). The prognostic value of hematological and systemic inflammatory disorders in invasive bladder cancer. Curr Opin Urol.

[23] Buck I, Morceau F, Grigorakaki C, Dicato M, Diederich M (2009). Linking anemia to inflammation and cancer: the crucial role of TNFalpha. Biochem Pharmacol.

[24] Morceau F, Dicato M, Diederich M (2009). Pro-inflammatory cytokine-mediated anemia: regarding molecular mechanisms of erythropoiesis. Mediators Inflamm.

[25] Ikonomi P, Rivera CE, Riordan M, Washington G, Schechter AN, Noguchi CT (2000). Overexpression of GATA-2 inhibits erythroid and promotes megakaryocyte differentiation. Exp Hematol.

[26] Pinedo HM, Verheul HM, D’Amato RJ, Folkman J (1998). Involvement of platelets in tumour angiogenesis?. Lancet.

[27] Xia G, Kumar SR, Hawes D, Cai J, Hassanieh L, Groshen S, Zhu S, Masood R, Quinn DI, Broek D, Stein JP, Gill PS (2006). Expression and significance of vascular endothelial growth factor receptor 2 in bladder cancer. J Urol.

[28] Nieswandt B, Hafner M, Echtenacher B, Männel DN (1999). Lysis of tumor cells by natural killer cells in mice is impeded by platelets. Cancer Res.

[29] Zhang N, Zhang WJ, Cai HQ, Liu HL, Peng L, Li CH, Ye LY, Xu SQ, Yang ZH, Lou JN (2011). Platelet adhesion and fusion to endothelial cell facilitate the metastasis of tumor cell in hypoxia-reoxygenation condition. Clin Exp Metastasis.

[30] Erler JT, Cawthorne CJ, Williams KJ, Koritzinsky M, Wouters BG, Wilson C, Miller C, Demonacos C, Stratford IJ, Dive C (2004). Hypoxia-mediated down-regulation of Bid and Bax in tumors occurs via hypoxia-inducible factor 1-dependent and -independent mechanisms and contributes to drug resistance. Mol Cell Biol.

[31] Yang MH, Wu MZ, Chiou SH, Chen PM, Chang SY, Liu CJ, Teng SC, Wu KJ (2008). Direct regulation of TWIST by HIF-1alpha promotes metastasis. Nat Cell Biol.

[32] Leo C, Giaccia AJ, Denko NC (2004). The hypoxic tumor microenvironment and gene expression. Semin Radiat Oncol.

[33] Hajian-Tilaki K (2013). Receiver Operating Characteristic (ROC) Curve Analysis for Medical Diagnostic Test Evaluation. Caspian J Intern Med.

