# Supplementary material for: Preoperative hemoglobin-platelet ratio can significantly predict progression and mortality outcomes in patients with T1G3 bladder cancer undergoing transurethral resection of bladder tumor
Source: Oncotarget. 2018 Jan 3;9(26):18627–36. doi: 10.18632/oncotarget.23896 (PMC5915098; doi:10.18632/oncotarget.23896)
Supplement: Supplementary file 1 [file oncotarget-09-18627-s001.pdf]

Preoperative hemoglobin-platelet ratio can significantly predict progression and mortality outcomes in patients with T1G3 bladder cancer undergoing transurethral resection of bladder tumor

SUPPLEMENTARY MATERIALS

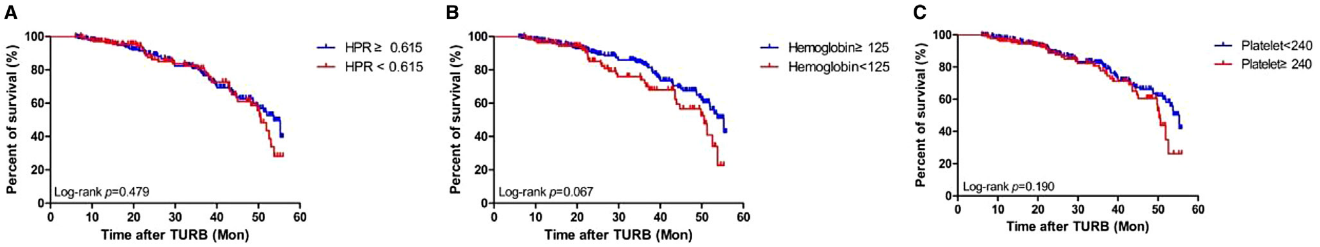

Supplementary Figure 1: Kaplan-Meier survival curves for comparing recurrence-free according to the preoperative status of (**A**) hemoglobin-platelet ratio (HPR), (**B**) Hemoglobin, (**C**) Platelet counts, respectively in the T1G3 bladder cancer patients who underwent transurethral resection of the bladder (TURB). Statistical differences were compared between the two groups by using the log-rank test
